# Influence of Previous Mental State on Psychological Outcomes of Spanish Out-of-Hospital Professionals during the COVID-19 Pandemic

**DOI:** 10.3390/ijerph20043574

**Published:** 2023-02-17

**Authors:** María Molina-Oliva, Rafael Martín-Sánchez, Elena Pastor-Benito, Raúl Soto-Cámara, Rosa M. Cárdaba-García, Israel John Thuissard, Juan José Fernández-Domínguez, María Paz Matellán-Hernández, Susana Navalpotro-Pascual, Almudena Morales-Sánchez

**Affiliations:** 1 Emergency Medical Service of Castilla y León–Sacyl, 47007 Valladolid, Spain; 2 Red de Investigación de Emergencias Prehospitalarias (RINVEMER), Sociedad Española de Urgencias y Emergencias (SEMES), 28020 Madrid, Spain; 3 Department of Nursing, Faculty of Nursing, University of Valladolid, 47005 Valladolid, Spain; 4 Emergency Medical Service of Madrid–SUMMA 112, 28045 Madrid, Spain; 5 Department of Health Sciences, University of Burgos, 09001 Burgos, Spain; 6 Department of Medicine, Faculty of Biomedical and Health Sciences, European University of Madrid, 28670 Madrid, Spain; 7 Emergency Service, HLA Moncloa University Hospital, 28080 Madrid, Spain; 8 Department of Nursing, Faculty of Medicine, Autonomous University of Madrid, 2029 Madrid, Spain

**Keywords:** COVID-19, health personnel, emergency medical services, psychological stress, anxiety, depression, self-efficacy, psychotropic drug, psychotherapy

## Abstract

This study aimed to describe factors relating to the psychological distress of healthcare workers (HCWs) in Spanish out-of-hospital emergency medical services (EMS), according to the previous or non-use of psychotropic drugs or psychotherapy. A multicentre, cross-sectional descriptive study was designed. The study population were all physicians, nurses, and emergency medical technicians (EMTs) working in any Spanish out-of-hospital EMS between February and April 2021. The main outcomes were the levels of stress, anxiety, depression, and self-efficacy, assessed by DASS-21 and G-SES. Differences in levels of stress, anxiety, depression, and self-efficacy, according to sex, age, previous use of psychotropic drug or psychotherapy, work experience, professional category, type of work, and modification of working conditions were measured using the Student’s *t*-test for independent samples, one-way ANOVA, Pearson’s correlation, or 2-factor analysis of covariance. A total of 1636 HCWs were included, of whom one in three had severe mental disorders because of the pandemic. The interaction of the previous or non-use of psychotropic drugs or psychotherapy with the rest of the factors considered did not modify the levels of stress, anxiety, depression, and self-efficacy. However, HCWs with a history of psychotropic drug or psychotherapy use had a more intense negative emotional response and lower self-efficacy, regardless of their sex, professional category, type of work, or change in the working conditions. These HCWs are considered particularly vulnerable to the development or recurrence of new disorders or other comorbidities; therefore, the implementation of monitoring and follow-up strategies should be a priority.

## 1. Introduction

Since the first case of coronavirus disease (COVID-19) was identified in Wuhan (China) in December 2019, the rapid worldwide spread of the SARS-CoV-2 virus has had relevant economic, social, and health repercussions [1].

To face this new scenario, the World Health Organization (WHO) declared the resulting situation as a “serious public health problem that all countries had to address” [2]. For this purpose, most governments implemented different measures, such as social distancing or forced home confinement, which have been relaxed or tightened depending on the evolution of the pandemic [1,3].

During this period, healthcare workers (HCWs) from out-of-hospital Emergency Medical Services (EMSs) have worked hard, becoming one of the main providers of care [4,5,6]. EMS is defined as “a comprehensive system which provides the arrangements of personnel, facilities, and equipment for the effective, coordinated and timely delivery of health and safety services to victims of sudden illness or injury” [7,8]. It has a coordinated notification mechanism for activation, which people must call as soon as an urgent or emergent situation is detected. After analyzing the patient’s needs, the EMS personnel assign an immediate response, which can be executed without mobilizing any resources or by moving its mobile care devices to the scene of the incident to act in situ, or to transfer the patient to the nearest health centre [8]. Its main objective is to prevent harmless mortality and long-term morbidity [9].

Out-of-hospital HCWs have often had to face unfavourable situations and to adapt their work conditions to the epidemiological scenario existing at any given time, including long working hours without rest periods, lack of approved personal protective equipment, ethical dilemmas in decision making or the use of unclear and constantly changing protocols [10,11]. The continuation of this situation over time, together with the fear of contagion and the social discrimination they often suffered, have had a negative impact on their behaviour, mood, and mental health [12,13]. In this regard, the COVID-19 pandemic is considered an event that favours the occurrence of post-traumatic stress symptoms among HCWs [14]. In this regard, several studies have observed a higher prevalence of these symptoms among those HCWs with poorer physical health and higher levels of anxiety and depression [15,16].

In general, HCWs have adopted multiple adaptative interventions and coping strategies to promote high self-efficacy perceptions, avoid maladaptive responses and reduce the risk of the mid-to-long-term associated pathologies [17]. The strategy used more frequently by the out-of-hospital HCWs is “stop unpleasant emotions and thoughts” [15]. A higher self-perception of emotional intelligence has been related to stronger levels of self-efficacy and engagement, thereby leading to a higher performance overall [18].

Some HCWs have used psychotropic drugs or psychotherapy [16,19,20], increasing their already high consumption in this group for other personal situations or circumstances [21,22]. The existing shortcomings of the different health systems in the care and follow-up of mental health problems have become evident during the pandemic and have hindered access to these treatments [23]. Despite this, significant efforts have been made to adapt mental health care to the demands arising from COVID-19 [24]. The impact of the pandemic on the most vulnerable HCWs, such as those with any previous mental disorder or with a history of psychotropic drug or psychotherapy use, has been poorly studied [25,26,27], especially among HCWs working in out-of-hospital EMSs. For this reason, this study aimed to describe factors relating to the levels of stress, anxiety, depression, and self-efficacy of HCWs in Spanish out-of-hospital Emergency Medical Services (EMSs), according to previous or non-use of psychotropic drugs or psychotherapy.

## 2. Materials and Methods

### 2.1. Study Design—Participants

A multicentre, cross-sectional, descriptive study was designed. The study population were all physicians, nurses, and emergency medical technicians (EMTs) working in any Spanish public or private out-of-hospital EMS between February and April 2021.

This study is part of a larger research project that, based on a mixed methodology, analyses the psychological impact of the COVID-19 pandemic on HCWs in Spanish out-of-hospital EMSs, while exploring the experiences of these workers and their meanings.

### 2.2. Procedure—Data Collection

Based on the voluntary nature of the study, the selection of participants was performed by non-probabilistic convenience snowball sampling. The invitation letter to the out-of-hospital HCWs to participate in this study was sent via email by the Prehospital Emergency Research Network (RINVEMER) of the Spanish Society of Emergency Medicine (SEMES) and by the managers of the different out-of-hospital EMSs. This letter informed of the main characteristics and objectives of the study, highlighting its anonymous and voluntary nature. Its final part included a link to the online questionnaire used for data collection, located on the e-Encuesta^®^ platform. To guarantee the anonymity of the participants, no personal data, which could allow their identification, were collected. The participants accessed and completed the questionnaire between 1 February and 30 April 2021. The time required to complete the questionnaire was approximately 12 min.

The completed return of the questionnaire implied the person’s informed consent to participate in the study. However, at any time, they could withdraw from the study without giving any reason.

The research protocol received a favourable report from the Institutional Review Board of the Valladolid East Health Area (Protocol code: PI20-2052) and was conducted following the ethical principles of the Helsinki Declaration and its successive revisions [28]. This study complied with the guidelines for reporting observational studies included in the STROBE initiative [29].

### 2.3. Main Outcomes—Instruments

The levels of stress, anxiety, depression, and self-efficacy of the out-of-hospital HCWs were the main outcomes of the study. The following instruments and questionnaires were used for their assessment:

- *The Depression Anxiety Stress Scale (DASS-21)*, was created by Lovibond et al. [30] and validated in the Spanish population by Bados et al. [31]. Using this scale, the person evaluates the frequency with which they have presented different symptoms associated with a negative emotional state in the previous two weeks through 21 items structured in three subscales: stress (tension, irritability, nervousness, impatience, agitation, and negative affect), anxiety (physiological activation, musculoskeletal symptoms, and subjective sensation of anxiety), and depression (hopelessness, dysphoria, sadness, anhedonia, low self-esteem, and low positive affect). Samples of items included on this scale are the following: “I felt that I was using a lot of nervous energy” (stress), “I was worried about situations in which I might panic and make a fool of myself” (anxiety), or “I felt that I had nothing to look forward to” (depression). A 4-point Likert-type scale is used, where 0 corresponds to never and 3 to always. In each subscale, the total score is obtained by adding the points of each item and multiplying it by 2. The score of the subscales ranges between 0 and 42, so the higher the value, the greater the degree of symptomatology. Similarly, this score can be categorized as normal, mild, moderate, severe, or extremely severe [30]. It has good discriminant validity in screening for mental disorders, with good psychometric properties [32].
- *The General Self-Efficacy Scale (G-SES)*, was created by Baessler et al. [33] and validated in the Spanish population by Sanjuán et al. [34]. Using this scale, the person’s perception of their ability to adequately handle different stressful situations is assessed through 10 items. Items included on this scale are the following: “I can always manage to solve difficult problems if I try hard enough” or “If someone opposes me, I can find the means and ways to get what I want”. A 10-point Likert-type scale is used, where 1 corresponds to never and 10 to always. The total score is obtained by adding the points of each item, ranging from 10 to 100. Therefore, the higher the score, the higher the level of perceived self-efficacy. It has good psychometric properties, with a predictive ability on coping styles and an internal consistency of 0.87 [33,34].

Other variables were also collected through an ad hoc questionnaire: sociodemographic (sex and age), clinical (previous use of psychotropic drugs or psychotherapy), and occupational (professional category, previous work experience, type of work, and modifications in working conditions)

### 2.4. Statistical Analysis

Descriptive analyses were conducted to describe the sample. Categorical variables were summarized as absolute frequencies and percentages, while continuous variables were expressed in terms of mean and standard deviation (SD). The Kolmogorov–Smirnov test was used to assess the compliance of normality criteria of the continuous variables; for cases which did not follow a normal distribution, the criteria proposed by Blanca et al. were considered [35]. Differences between previous or non-use of psychotropic drugs or psychotherapy on the levels of stress, anxiety, depression, and self-efficacy, according to sex, age, work experience, professional category, type of work, and modification of working conditions were measured using the Student’s *t*-test for independent samples, one-way ANOVA, or Pearson’s correlation depending on the nature of the variables. For multiple comparisons, post hoc tests were corrected by Bonferroni’s adjustment. In addition, to know if the previous use of psychotropic drugs or psychotherapy was a determining factor in the psychological impact of each of the variables, a 2-factor analysis of covariance (study variables x previous use of psychotropic drugs or psychotherapy) was performed. Effect sizes were calculated using partial eta squared (η^2^ *p*) and interpreted according to the following criteria: if 0 ≤ η^2^ *p* < 0.05, there is no effect; if 0.05 ≤ η^2^ *p* < 0.26, this effect is minimal; if 0.26 ≤ η^2^ *p* < 0.64, this effect is moderate; and if η^2^ *p* ≥ 0.64, this effect is strong [36]. Statistical significance was considered if *p* < 0.05. Statistical analysis was performed with SPSS software version 28.0 (IBM-Inc, Chicago, IL, USA).

## 3. Results

A total of 1636 HCWs voluntarily agreed to participate in the study; 50.43% (*n* = 811) were women, with a mean age of 43.51 years (SD ± 9.98). EMTs were the most represented professional category (*n* = 739), followed by nurses (*n* = 441) and physicians (*n* = 438). During the pandemic, 54.43% (*n* = 258) had to change their working conditions; most worked in direct patient care on the front line (*n* = 1415; 86.49%). Their mean work experience in the out-of-hospital EMSs was 15.22 years (SD ± 9.17). One in five participants reported having taken psychotropic drugs or psychotherapy at some point before the pandemic began. The distribution of their descriptive characteristics, based on the use or non-use of psychotropic drugs or psychotherapy, is summarized in Table 1.

Concerning the mental health of these professionals, 37.22% (*n* = 609), 39.49% (*n* = 646), and 30.50% (*n* = 499) presented levels of stress, anxiety, and depression categorized as severe or extremely severe. The mean score obtained in stress, anxiety, and depression were 20.62 (SD ± 11.06), 14.00 (SD ± 11.15), and 15.75 (SD ± 11.63), respectively. Statistically higher values were observed in HCWs with a personal history of taking psychotropic drugs or psychotherapy. Regarding self-efficacy, the mean score was 70.74 (SD ± 15.77), with greater levels in HCWs who had not previously used psychotropic drugs or psychotherapy (Table 2).

Both men and women who had needed to use psychotropic drugs or psychotherapy before the start of the pandemic reported higher levels of stress, anxiety, and depression, as well as lower values of self-efficacy. On the other hand, men who had not previously required psychotropic drugs or psychotherapy presented lower stress than women who had not required them either. The interaction of sex and previous use of psychotropic drugs or psychotherapy did not affect the psychological variables analyzed (Table 3).

Comparing professional categories, EMTs reported worse negative emotional states regardless of whether they had previously used psychotropic drugs or psychotherapy. The stress, anxiety, and depression levels of physicians, nurses, and EMTs were higher if they had needed to take psychotropic drugs or psychotherapy in the past; furthermore, a lower degree of competence to cope adequately with different stressful life situations was observed in these HCWs. The professional category and previous use of psychotropic drugs or psychotherapy combination did not influence the mean scores on the DASS-21 and the G-SES (Table 4).

HCWs with a personal history of using psychotropic drugs or psychotherapy showed more severe levels of stress, anxiety, and depression, as well as less confidence in their ability to achieve the proposed outcomes, regardless of whether they worked on the frontline in direct contact with the patient or at the coordinating centre answering emergency calls. When the interaction of both variables was analyzed, no influence was observed on the psychological parameters studied (Table 5).

When considering the modification or non-modification of working conditions, greater degrees of stress, anxiety, depression, and lower self-efficacy were observed in HCWs who had used psychotropic drugs or psychotherapy on a previous occasion. On the other hand, more severe levels of stress, anxiety, and depression were observed in HCWs who were forced to change their work schedule, dedication, or location, in the same group of use or non-use of psychotropic drugs or psychotherapy before the start of the pandemic. When the psychological impact of the COVID-19 pandemic was analyzed, considering the need or not for changes in working conditions, it was concluded that the use or non-use of psychotropic drugs or psychotherapy was not a determining factor (Table 6).

Both the age of HCWs and work experience in out-of-hospital EMS were indirectly and weakly correlated with levels of stress, anxiety and depression in HCWs who had not used psychotropic drugs or psychotherapy prior to the onset of the pandemic (Table 7).

## 4. Discussion

This study was proposed to analyze the relationship between the mental health and self-efficacy of HCWs in Spanish out-of-hospital EMSs during the COVID-19 pandemic and several sociodemographic and occupational variables, according to the previous or non-consumption of psychotropic drugs or psychotherapy. The levels of stress, anxiety, depression, and self-efficacy of the HCWs have not been modified by the interaction of prepandemic history of the use or non-use of psychotropic drugs or psychotherapy with the rest of the factors considered.

The findings of this study show that one in three HCWs reported having psychopathological levels of stress, anxiety, and depression, which is higher than that observed in other care settings [37,38,39,40,41,42]. This high prevalence should be considered a warning sign of possible negative psychosocial consequences from the acute phase of the pandemic, such as burnout or post-traumatic stress [15,43]. Although HCWs in the out-of-hospital setting are highly trained to respond to unpredictable and potentially traumatic situations, having become one of the first providers of health care to patients with suspected or confirmed signs of COVID-19 has been cited as one of the major causes of their increased psychological distress [13,44]. However, other authors argue that these HCWs have lower levels of stress, anxiety, and depression than those working in inpatient units [26,45]. This lower emotional burden may be because these HCWs perceived their work during the pandemic as a continuation of their regular work with specific self-protection measures, and their actions are limited to the initial phases of patient care [26,45].

Specialized psychological or psychiatric treatment use has traditionally been considered an indirect marker of a person’s mental health [46,47]. This study shows that around 20% of HCWs already used psychotropic drugs or psychotherapy before the onset of the pandemic. This figure is similar to that obtained in other studies [48,49] and slightly higher than the 10.7% reported by the Spanish Health Authorities for the general population [50]. This greater use among HCWs may be due to the high workloads they are subjected to and their ease of access to self-prescribe these treatments [51]. Moreover, during the different phases of the pandemic, their use has increased exponentially due to multiple factors, including the fear of catching or transmitting the disease to their relatives, the lack of material/human resources or the lack/excess of information [19,20]. However, it should be noted that there is an under-diagnosis of mental pathology among HCWs, which may be related to the reluctance to ask for help, fear of social stigmatization of the person, suspicion of non-confidentiality about their clinical data, or possible negative repercussions on their professional career [19,52]. HCWs with a history of psychotropic drug use or psychotherapy had a more intense negative emotional response and lower self-efficacy, regardless of their sex, professional category, whether they worked on the front line or not, or whether they had to change their working conditions or not. This result may be because HCWs with a previous mental disease are not in the best psycho-emotional state to face disruptive situations, such as that derived from the pandemic, which takes the person out of their comfort zone and requires a continuous ability to adapt [27,53,54,55,56,57]. Along the same lines, several authors have concluded that this association may be influenced by the high recurrence rate and chronic nature of most mental diseases [26,58,59]. In addition, having suffered previous mental disorders has been considered a predisposing factor for the development, persistence, or recurrence of certain mental illnesses or other types of comorbidities [26,27,38,53,60,61,62,63,64]. In any case, based on these findings, the importance of designing and generalizing new strategies for the control and monitoring of the most vulnerable HCWs, through the implementation of programs for the prevention and early detection of mental disorders and the adoption of appropriate support measures, is emphasized [26,27,65]. In this way, their mental health is prevented from worsening, and consequently, the effectiveness and quality of the care they provide are not compromised [25]. The acceptance of these measures by HCWs at risk makes them a focus for further research.

Among HCWs who reported no previous use of psychotropic drugs or psychotherapy, women had higher levels of stress, anxiety, and depression, while men had higher self-efficacy scores. In this regard, the gender bias in women’s health care is noteworthy. On many occasions, non-specific pain or anodyne symptoms, which do not fit a specific clinical picture, are diagnosed as psycho-emotional disorders and treated with psychotropic drugs or psychotherapy [66,67]. In these cases, the requirements of good practice, which recommend ruling out organic causes before blaming symptoms on a mental health problem, are not met [67,68]. This gender discrimination arises from the biologist, androcentric, and patriarchal vision of health [69]. The scientific literature shows that women are subject to higher risk factors for psycho-emotional disorders. Among the mentioned factors, during the pandemic, the difficulty in reconciling work and family life, the traditional assumption of the role of primary caregiver at home, the lack of adequate support systems, their greater empathic capacity in the provision of care or longer working hours stand out [70,71,72]. In addition, women tend to develop emotion-focused coping strategies more often than men, which are less effective in the face of adverse situations [59,73].

In terms of age, as findings in other studies [27,74,75], younger HCWs who had not previously used psychotropic drugs or psychotherapy showed higher levels of all psychological symptomatology assessed. During the pandemic, many of these workers faced an unfamiliar, unpredictable, and complex situation for the first time, regardless of their professional role or work setting [76]. As a result, they have less self-confidence and psychological resilience, which leads to a higher degree of uncertainty about how to act and a poorer adaptation to the stressors that may be presented to them [77]. In addition, sudden changes in lifestyles and disruption to regular social activities increased the negative impact of the pandemic on the mental health of younger HCWs to a greater extent [78]. Some studies have identified that older HCWs are more concerned about their safety, while younger HCWs are more concerned about infecting their families [79]. Thus, age is a factor that favours the development of coping strategies and resilience due to exposure to multiple stressors over time. It results in better emotional management and less anxious–depressive symptomatology [80].

Related to age, HCWs with less work experience in out-of-hospital care and no previous use of psychotropic drugs or psychotherapy had higher levels of stress, anxiety, and depression. Generally, these workers are younger, have fewer practical skills to manage complex situations or conflicts with patients, do not have robust social supports or job security, and feel more shocked and impressionable by settings that are perhaps more expected of their older and more experienced colleagues [37,81]. This result might be a consequence of the acquisition of resilience and the development of adaptive coping mechanisms through years of work [82,83].

As concluded by other authors [52,57,84,85], EMTs were the professional category most notably affected by the pandemic, regardless of whether or not they had previously used psychotropic drugs or psychotherapy. Possible reasons for this finding include their poorer working conditions and the higher risk of infection due to the inadequate use of personal protective equipment with patients who did not show respiratory symptoms [52,57,86]. In contrast, doctors and nurses tend to be more resistant to somatisation because of their achievements, previous work experience, or self-awareness. In addition, the habitual use of coping strategies based on intellectualisation and denial protects them against the development of psychopathological levels of stress, anxiety, and depression [86,87]. These results contrast with those obtained in other studies, which place nurses or physicians as the most affected HCWs [88,89,90,91,92]. This lack of unanimity in the results may be due to heterogeneity in the structure, organisation, and functioning of worldwide out-of-hospital EMSs.

During the COVID-19 pandemic, out-of-hospital EMSs responded to a growing number of phone demands for urgent health care, making their out-of-hospital HCWs one of the main care providers [5,6,93]. However, to achieve this, it has been necessary to continuously change their working conditions to cope with the epidemiological situation at any given time [92,94]. These changes, together with a social context marked by restrictions and fear, increased the vulnerability to more severe psycho-emotional disorders in the HCWs studied, regardless of whether or not they had previously used psychotropic drugs or psychotherapy. On the one hand, the reassignment of HCWs to other units dedicated exclusively to the care and transport of patients with suspected symptoms or confirmed cases of COVID-19 has been one of the measures adopted by most out-of-hospital EMSs [93]. The adaptation to this new work context, together with the assumption of new tasks that were not part of their usual duties, the lack of theoretical-practical training, the continuous changes in guidelines and protocols or the use of unapproved personal protective equipment, have placed additional mental burdens on displaced HCWs [10,11,26,74,94]. These factors have also favoured the emergence of fear and uncertainty among HCWs, not only because of the increased risk of contagion but also because of their possible involvement in affecting patient safety [93]. In addition, this situation has had an impact on interprofessional collaboration and may have induced the occurrence of conflicts with non-regular colleagues [83]. Comparing these results with those obtained in other care settings, reassigned HCWs have shown less psychological distress, higher levels of resilience, and more social support, which may be due to their ability to respond to unpredictable and potentially traumatic situations [45]. On the other hand, the increasing number of infections among HCWs and the obligation to quarantine after contact with an infected patient have led to longer working hours and reduced rest periods for HCWs who do not fall ill and continue to work. [45,93]. This situation has also increased the negative effect of prolonged exposure to critical care scenarios on the mental health of HCWs [25]. Failure to recognise the crucial role of organisational and relational work factors in the psycho-emotional state of HCWs may unfairly blame symptomatic individuals for not showing sufficient resilience. To avoid it, healthy work environments need to foster an improved patient safety culture and wider changes to implemented health policies [95,96].

In this study, working in direct contact with patients, where the unpredictability of cases or the probability of contagion is high, was not related to a greater susceptibility of HCWs to develop negative psycho-emotional responses, contrary to the results obtained by other authors [19,27,97]. This lack of statistical significance may be because frontline out-of-hospital HCWs have perceived their actions as the continuity of their habitual procedures and activities, although with higher levels of self-protection and security, and because HCWs in the emergency coordination centre have been under increased pressure due to the volume of emergency calls they have to deal with. In both cases, they are more accustomed to living with potentially stressful experiences and showing fewer negative responses in the face of challenging situations [98].

Workplace training, promotion of self-care, peer support, and psycho-affective education are some of the strategies implemented to promote the mental health of these HCWs during the COVID-19 pandemic [99]. Most of them have been developed in a rapidly evolving situation where their clinical needs have been prioritized over research methods. The heterogeneity of these programs, their no maintenance over time, and the lack of standardized protocols have made it impossible to determine what strategy offers higher benefits [100]. Further research is needed to analyze how best to support the mental well-being of HCWs, taking into account organizational, social, personal, and psychological factors.

The strengths of this study include using institutional mailing lists as a reliable sampling framework, its multicentric character by collecting data from most Spanish out-of-hospital EMSs, and the availability of representative data from many HCWs. These strengths support the robustness and relevance of the findings. Nevertheless, the study has some limitations that deserve careful consideration. The cross-sectional nature of this study precludes the inference of any causal impact of the COVID-19 pandemic on the mental health of HCWs. In addition, data collection lasted 12 weeks, offering a static image of the situation, only valid for that specific moment. The selection of participants through non-probabilistic snowball sampling may have induced a self-selection bias. Consequently, HCWs who were particularly sensitive to the issue or those with a greater affectation were more likely to participate in the study. However, it is also probable that the most stressed HCWs did not have time to answer the questionnaire. In order to improve their representativeness, the observed data have been carefully weighted to exactly reproduce the distribution by gender, age, and professional category of the HCWs of the study population. Instruments used to assess mental disorders are based on self-reports by HCWs rather than clinical diagnoses. However, the sensitivity and specificity for the stress, anxiety, and depression score cutoffs have proven to be acceptable [30,32]. Moreover, these instruments are among the most frequently used in epidemiologic studies, which allow for the comparability of results. The small number of studies on this topic in the out-of-hospital setting hinders the comparison and contrast of the results obtained.

This study has important implications for future research, clinical practice, and public health. The use of psychotropic drugs or psychotherapy before an adverse event must be considered a risk factor for adverse mental outcomes in out-of-hospital HCWs. In this way, the professionals particularly vulnerable to deterioration of their mental health could be detected and their assistance prioritized. These results might be taken into account in the planning and implementation of future programs for the prevention and early detection of mental health disorders, as well as in the development of public health initiatives and psychological interventions to help build resilience in these HCWs.

## 5. Conclusions

The HCWs from Spanish out-of-hospital EMSs present high degrees of stress, anxiety, depression, and medium degrees of self-efficacy. Those HCWs with a history of psychotropic drug or psychotherapy use have a more intense negative emotional response and lower self-efficacy, regardless of their sex, professional category, type of work, or change in the working conditions. These HCWs are considered particularly vulnerable to the development or recurrence of new disorders or other comorbidities, so the implementation of monitoring and follow-up strategies should be a priority.

## Figures and Tables

**Table 1:** Descriptive characteristics of the sample based on the previous use of psychotropic drugs or psychotherapy.

	Previous Use of Psychotropic Drugs or Psychotherapy	
	Yes 296 (18.09)	No 1340 (81.91)
**Sex**		
Male	120 (7.33)	691(42.24)
Female	176 (10.76)	649 (39.67)
**Age** (years)	42.99 ± 9.68	43.62 ± 10.04
**Professional category**		
Physician	75 (4.58)	363 (22.19)
Nurse	87 (5.32)	354 (21.64)
EMT	129 (7.88)	610 (37.29)
Other	5 (0.31)	13 (0.79)
**Frontline work**		
Yes	250 (15.28)	1165 (71.21)
No	46 (2.81)	175 (10.70)
**Work experience in out-of-hospital EMS** (years)	15.04 ± 8.94	15.26 ± 9.23
**Modifications in working conditions**		
Yes	194 (11.86)	689 (42.12)
No	102 (6.23)	651 (39.79)

Abbreviation: EMT—Emergency Medical Technicians. EMS—Emergency Medical Service.

**Table 2:** Level of stress, anxiety, depression, and self-efficacy according to previous use of psychotropic drugs or psychotherapy.

	Previous Use of Psychotropic Drugs or Psychotherapy		*p*-Value
	Yes	No	
**Stress**	27.26 ± 9.763	19.15 ± 10.79	<0.001
**Anxiety**	20.28 ± 11.93	11.51 ± 10.32	<0.001
**Depression**	21.50 ± 11.58	14.48 ± 10.62	<0.001
**Self-Efficacy**	66.20 ± 18.64	71.75 ± 14.89	<0.001

**Table 3:** Level of stress, anxiety, depression, and self-efficacy according to sex and previous use of psychotropic drugs or psychotherapy.

Previous Use of Psychotropic Drugs or Psychotherapy	Sex		*p*-Value (Sex × Psychotropic Drugs or Psychotherapy)	η^2^ *p*
	Male	Female		
*Stress*				
Yes	26.86 ± 10.20 ***	27.68 ± 9.27 ***	0.052	0.002
No	17.48 ± 10.62 ^$$$,^***	20.97 ± 10.67 ^$$$,^***		
*Anxiety*				
Yes	19.71 ± 11.89 ***	20.78 ± 11.91 ***	0.239	0.001
No	10.22 ± 10.01 ^$$$,^***	12.91 ± 10.47 ^$$$,^***		
*Depression*				
Yes	21.16 ± 11.26 ***	21.85 ± 11.73 ***	0.230	0.001
No	13.34 ± 10.27 ^$$$,^***	15.72 ± 10.85 ^$$$,^***		
*Self-Efficacy*				
Yes	66.33 ± 19.89 **	66.44 ± 17.35 **	0.237	0.001
No	72.85 ± 14.38 ^$$,^**	70.55 ± 15.31 ^$$,^**		

Values are expressed as mean ± standard deviation. ^$$^ *p* < 0.01 between sexes in the same group of previous use of psychotropic drugs or psychotherapy. ^$$$^ *p* < 0.001 between sexes in the same group of previous or the use of psychotropic drugs or psychotherapy. ** *p* < 0.01 between previous use of psychotropic drugs or psychotherapy in the same sex group; *** *p* < 0.001 between previous use of psychotropic drugs or psychotherapy in the same sex group.

**Table 4:** Level of stress, anxiety, depression, and self-efficacy according to professional categories and previous use of psychotropic drugs or psychotherapy.

Previous Use of Psychotropic Drugs or Psychotherapy	Professional Categories				*p*-Value (Categories × Psychotropic Drugs or Psychotherapy)	η^2^ *p*
	Physician	Nurse	EMT	Other		
*Stress*						
Yes	27.15 ± 9.49 ^$$$,^***	24.18 ± 11.64 ^$$$,^***^,b^	29.47 ± 8.01 ^$$$,^***^,b^	18.16 ± 12.08 ^$$$,^*	0.161	0.003
No	18.33 ± 10.91 ^$$$,^***^,a^	18.40 ± 10.85 ^$$$,^***^,b^	20.20 ± 10.91 ^$$$,^***^,a,b^	14.31 ± 10.90 ^$$$,^*		
*Anxiety*						
Yes	18.16 ± 12.08 ^$$$,^***^,a^	17.47 ± 13.53 ^$$$,^***^,b^	23.58 ± 9.85 ^$$$,^***^,a,b^	16.00 ± 11.49 ^$$$^	0.266	0.002
No	9.58 ± 9.96 ^$$$,^***^,a^	10.35 ± 9.45 ^$$$,^***^,b^	13.37 ± 10.71 ^$$$,^***^,a,b^	9.69 ± 10.48 ^$$$^		
*Depression*						
Yes	22.21 ± 10.8 ^$$$,^***	10.50 ± 12.86 ^$$$,^***^,b^	23.30 ± 10.14 ^$$$,^***^,b^	16.40 ± 10.24 ^$$$^	0.134	0.003
No	12.98 ± 10.45 ^$$$,^***	13.65 ± 10.18 ^$$$,^***	15.97 ± 10.84 ^$$$,^***	9.08 ± 6.81 ^$$$^		
*Self-Efficacy*						
Yes	68.31 ± 18.33 *	63.74 ± 20.12 ***	66.70 ± 17.78 ^***^	64.80 ± 19.24	0.398	0.002
No	72.47 ± 14.83 *	71.59 ± 14.49 ***	71.31 ± 15.21 ^***^	76.54 ± 11.54		

Values are expressed as mean ± standard deviation. Abbreviation: EMT—Emergency Medical Technicians. ^$$$^ *p* < 0.001 between professional categories in the same group of previous use of psychotropic drugs or psychotherapy. * *p* < 0.05 between previous use of psychotropic drugs or psychotherapy in the same professional category; *** *p* < 0.001 between previous use of psychotropic drugs or psychotherapy in the same professional category. ^a,b^ *p* < 0.05 in the post hoc analysis (Bonferroni test).

**Table 5:** Level of stress, anxiety, depression, and self-efficacy according to the type of work and previous use of psychotropic drugs or psychotherapy.

Previous Use of Psychotropic Drugs or Psychotherapy	Frontline Work		*p*-Value (Care Work × Psychotropic Drugs or Psychotherapy)	η^2^ *p*
	Yes	No		
*Stress*				
Yes	27.30 ± 9.54 ***	27.00 ± 10.99 ***	0.307	0.001
No	18.94 ± 10.74 ***	20.58 ± 11.01 ***		
*Anxiety*				
Yes	20.32 ± 12.06 ***	20.09 ± 11.37 ***	0.764	0.001
No	11.46 ± 10.33 ***	11.81 ± 10.30 ***		
*Depression*				
Yes	21.64 ± 11.48 ***	21.70 ± 12.23 ***	0.789	0.001
No	14.38 ± 10.57 ***	15.31 ± 10.94 ***		
*Self-Efficacy*				
Yes	67.14 ± 18.16 ***	61.11 ± 20.56 ***	0.085	0.002
No	71.91 ± 15.03 ***	70.70 ± 13.91 ***		

Values are expressed as mean ± standard deviation. *** *p* < 0.001 between previous use of psychotropic drugs or psychotherapy in the same type of work group.

**Table 6:** Level of stress, anxiety, depression, and self-efficacy according to modifications in working conditions and previous use of psychotropic drugs or psychotherapy.

Previous Use of Psychotropic Drugs or Psychotherapy	Modifications in Working Conditions		*p*-Value (Modifications × Psychotropic Drugs or Psychotherapy)	η^2^ *p*
	Yes	No		
*Stress*				
Yes	28.42 ± 11.84 ^$$$,^***	25.04 ± 9.59 ^$$$,^***	0.622	0.001
No	20.46 ± 10.53 ^$$,^***	17.77 ± 10.89 ^$$,^***		
*Anxiety*				
Yes	21.78 ± 11.84 ^$$$,^***	17.43 ± 10.37 ^$$$,^***	0.103	0.002
No	12.50 ± 10.37 ^$$,^***	10.46 ± 10.16 ^$$,^***		
*Depression*				
Yes	23.30 ± 11.64 ^$$,^***	18.02 ± 10.69 ^$$,^***	0.059	0.004
No	15.36 ± 10.58 ^$$$,^***	13.55 ± 10.59 ^$$$,^***		
*Self-Efficacy*				
Yes	64.98 ± 18.66 ***	68.52 ± 18.48 *	0.179	0.001
No	71.39 ± 14.92 ***	72.12 ± 14.86 *		

Values are expressed as mean ± standard deviation. ^$$^ *p* < 0.01 between modifications in working conditions in the same group of previous use of psychotropic drugs or psychotherapy. ^$$$^ *p* < 0.001 between modifications in working conditions in the same group of previous use of psychotropic drugs or psychotherapy use. * *p* < 0.05 between previous use of psychotropic drugs or psychotherapy in the same group of modifications in working conditions; *** *p* < 0.001 between previous use of psychotropic drugs or psychotherapy in the same group of modifications in working conditions.

**Table 7:** Level of stress, anxiety, depression, and self-efficacy according to age, EMS work experience and previous use of psychotropic drugs or psychotherapy.

	Previous Use of Psychotropic Drugs or Psychotherapy	Stress	Anxiety	Depression	Self-Efficacy
**Age**	Yes	0.050	−0.017	0.027	−0.058
	No	−0.179 ***	−0.153 ***	−0.137 ***	−0.030
**Work experience in out-of-hospital EMS**	Yes	−0.006	−0.048	−0.018	−0.033
	No	−0.167 ***	−0.158 ***	−0.133 ***	0.026

Values are expressed as Spearman’s correlation coefficient. Abbreviation: EMS—Emergency Medical Service. *** *p* < 0.001.

## Data Availability

Not applicable.

## References

[1] García-Iglesias J.J., Gómez-Salgado J., Martín-Pereira J., Fagundo-Rivera J., Ayuso-Murillo D., Martínez-Riera J.R., Ruiz-Frutos C. (2020). Impact of SARS-CoV-2 (COVID-19) on the Mental Health of Healthcare Professionals: A Systematic Review. Rev. Esp. Salud Publica.

[2] WHO Director-General’s Opening Remarks at the Media Briefing on COVID-19. 11 March 2020. World Health Organization (WHO). https://www.who.int/director-general/speeches/detail/who-director-general-s-opening-remarks-at-the-media-briefing-on-covid-19---11-march-2020.

[3] Canet-Juric L., Andrés M.L., del Valle M., López-Morales H., Poó F., Galli J.I., Yerro M., Urquijo S. (2020). A Longitudinal Study on the Emotional Impact Cause by the COVID-19 Pandemic Quarantine on General Population. Front. Psychol..

[4] Martínez-Riera J.R., Gras-Nieto E. (2021). Atención Domiciliaria y COVID-19. Home Care and COVID-19. Before, in and after the State of Alarm. Enferm Clin..

[5] Şan İ., Usul E., Bekgöz B., Korkut S. (2021). Effects of COVID-19 Pandemic on Emergency Medical Services. Int. J. Clin. Pract..

[6] Snooks H., Watkins A.J., Bell F., Brady M., Carson-Stevens A., Duncan E., Evans B.A., England L., Foster T., Gallanders J. (2021). Call Volume, Triage Outcomes, and Protocols during the First Wave of the COVID-19 Pandemic in the United Kingdom: Results of a National Survey. J. Am. Coll. Emerg. Physicians Open.

[7] Moore L. (1999). Measuring Quality and Effectiveness of Prehospital EMS. Prehosp. Emerg. Care.

[8] Barroeta Urquiza J., Boada Bravo N. (2011). Los Servicios de Emergencias y Urgencias Médicas Extrahospitalarias en España.

[9] Al-Shaqsi S. (2010). Models of International Emergency Medical Service (EMS) Systems. Oman Med. J..

[10] Alwidyan M.T., Oteir A.O., Trainor J. (2022). Working during Pandemic Disasters: Views and Predictors of EMS Providers. Disaster Med. Public Health. Prep..

[11] Ventura C., Gibson C., Collier G.D. (2020). Emergency Medical Services Resource Capacity and Competency Amid COVID-19 in the United States: Preliminary Findings from a National Survey. Heliyon.

[12] Soto-Cámara R., García-Santa-Basilia N., Onrubia-Baticón H., Cárdaba-García R.M., Jiménez-Alegre J.J., Reques-Marugán A.M., Molina-Oliva M., Fernández-Domínguez J.J., Matellán-Hernández M.P., Morales-Sanchez A. (2021). Psychological Impact of the COVID-19 Pandemic on Out-of-Hospital Health Professionals: A Living Systematic Review. J. Clin. Med..

[13] Ventura C.A.I., Denton E.E., David J.A., Schoenfelder B.J., Mela L., Lumia R.P., Rudi R.B., Haldar B. (2022). Emergency Medical Services Prehospital Response to the COVID-19 Pandemic in the US: A Brief Literature Review. Open Access Emerg. Med..

[14] Sanchez-Gomez M., Giorgi G., Finstad G.L., Urbini F., Foti G., Mucci N., Zaffina S., León-Perez J.M. (2021). COVID-19 Pandemic as a Traumatic Event and Its Associations with Fear and Mental Health: A Cognitive-Activation Approach. Int. J.Environ. Res. Public Health.

[15] Vagni M., Maiorano T., Giostra V., Pajardi D. (2020). Coping with Covid-19: Emergency Stress, Secondary Trauma and Self-Efficacy in Healthcare and Emergency Workers in Italy. Front. Psychol..

[16] Raudenská J., Steinerová V., Javůrková A., Urits I., Kaye A.D., Viswanath O., Varrassi G. (2020). Occupational burnout syndrome and Post-Traumatic Stress among Healthcare Professionals during the Novel Coronavirus Disease 2019 (COVID-19) Pandemic. Best. Pract. Res. Clin. Anaesthesiol..

[17] Bosmans G., Hofland H.W., De Jong A.E., Van Loey N.E. (2015). Coping with Burns: The Role of Coping Self-Efficacy in the Recovery from Traumatic Stress Following Burn Injuries. J. Behav. Med..

[18] Sanchez-Gomez M., Sadovyy M., Breso E. (2021). Health-Care Professionals Amid the COVID-19 Pandemic: How Emotional Intelligence May Enhance Work Performance Traversing the Mediating Role of Work Engagement. J. Clin. Med..

[19] Danet Danet A. (2021). Psychological Impact of COVID-19 Pandemic in Western Frontline Healthcare Professionals. A Systematic Review. Med. Clin..

[20] Kisely S., Warren N., McMahon L., Dalais C., Henry I., Siskind D. (2020). Occurrence, Prevention, and Management of the Psychological Effects of Emerging Virus Outbreaks on Healthcare Workers: Rapid Review and Meta-Analysis. BMJ.

[21] Purgato M., Gastaldon C., Papola D., van Ommeren M., Barbui C., Tol W.A. (2018). Psychological Therapies for the Treatment of Mental Disorders in Low- and Middle-Income Countries Affected by Humanitarian Crises. Cochrane Database Syst. Rev..

[22] Buitrago Ramírez F., Ciurana Misol R., Fernández Alonso M.D.C., Tizón J.L. (2021). COVID-19 Pandemic and Mental Health: Initial Considerations from Spanish Primary Health Care. Aten. Primaria.

[23] Zamorano S., Ausín B., González-Sanguino C., Castellanos M.Á., Saiz J., Abad A., Vaquero C., Muñoz M. (2022). Impacto del Covid-19 en la Salud Mental, Uso y Barreras en Atención Psicológica en España. Clínica Contemp..

[24] Moreno C., Wykes T., Galderisi S., Nordentoft M., Crossley N., Jones N., Cannon M., Correll C.U., Byrne L., Carr S. (2020). How Mental Health Care Should Change as a Consequence of the COVID-19 Pandemic. Lancet Psychiatry.

[25] Sobregrau Sangrà P., Aguiló Mir S., Castro Ribeiro T., Esteban-Sepúlveda S., García Pagès E., López Barbeito B., Pomar Moya-Prats J.L., Pintor Pérez L., Aguiló Llobet J. (2022). Mental Health Assessment of Spanish Healthcare Workers during the SARS-CoV-2 Pandemic. A Cross-Sectional Study. Compr. Psychiatry.

[26] Lasalvia A., Bonetto C., Porru S., Carta A., Tardivo S., Bovo C., Ruggeri M., Amaddeo F. (2020). Psychological Impact of COVID-19 Pandemic on Healthcare Workers in a Highly Burdened Area of North-East Italy. Epidemiol. Psychiatr. Sci..

[27] Alonso J., Vilagut G., Mortier P., Ferrer M., Alayo I., Aragón-Peña A., Aragonès E., Campos M., Cura-González I.D., Emparanza J.I. (2021). Mental Health Impact of the First Wave of COVID-19 Pandemic on Spanish Healthcare Workers: A Large Cross-Sectional Survey. Rev. Psiquiatr. Salud Ment..

[28] World Medical Association (2013). World Medical Association Declaration of Helsinki: Ethical Principles for Medical Research Involving Human Subjects. JAMA.

[29] Von Elm E., Altman D.G., Egger M., Pocock S.J., Gøtzsche P.C., Vandenbroucke J.P. (2008). The Strengthening the Reporting of Observational Studies in Epidemiology (STROBE) Statement: Guidelines for Reporting Observational Studies. Gac. Sanit..

[30] Lovibond P.F., Lovibond S.H. (1995). The Structure of Negative Emotional States: Comparison of the Depression Anxiety Stress Scales (DASS) with the Beck Depression and Anxiety Inventories. Behav. Res. Ther..

[31] Bados A., Solanas R., Andrés R. (2005). Psychometric Properties of the Spanish Version of Depression, Anxiety and Stress Scales (DASS). Psicothema.

[32] Mitchell M.C., Burns N.R., Dorstyn D.S. (2008). Screening for Depression and Anxiety in Spinal Cord Injury with DASS-21. Spinal Cord..

[33] Baessler J., Schwarcer R. (1996). Evaluación de la Autoeficacia: Adaptación Española de la Escala de Autoeficacia General. Ansiedad Estrés.

[34] Sanjuán Suárez P., Pérez García A.M., Bermúdez Moreno J. (2000). Escala de Autoeficacia General: Datos Psicométricos de la Adaptación para Población Española. Psicothema.

[35] Blanca M.J., Alarcón R., Arnau J., Bono R., Bendayan R. (2017). Non-Normal Data: Is ANOVA still a Valid Option?. Psicothema.

[36] Ferguson C.J. (2009). An Effect Size Primer: A Guide for Clinicians and Researchers. Prof. Psychol. Res. Pract..

[37] Dosil Santamaría M., Ozamiz-Etxebarria N., Redondo Rodríguez I., Jaureguizar Alboniga-Mayor J., Picaza Gorrotxategi M. (2021). Psychological Impact of COVID-19 on a Sample of Spanish Health Professionals. Rev. Psiquiatr. Salud Ment..

[38] Azizi M., Kamali M., Moosazadeh M., Aarabi M., Ghasemian R., Hasannezhad Reskati M., Elyasi F. (2021). Assessing Mental Health Status among Iranian Healthcare Workers in Times of the COVID-19 Pandemic: A Web-Based Cross-Sectional Study. Brain Behav..

[39] Juliana N., Mohd Azmi N.A.S., Effendy N., Mohd Fahmi Teng N.I., Azmani S., Baharom N., Mohamad Yusuff A.S., Abu I.F. (2022). Exploring the Associated Factors of Depression, Anxiety, and Stress among Healthcare Shift Workers during the COVID-19 Pandemic. Int. J. Environ. Res. Public Health.

[40] Chew N.W.S., Lee G.K.H., Tan B.Y.Q., Jing M., Goh Y., Ngiam N.J.H., Yeo L.L.L., Ahmad A., Ahmed Khan F., Napolean Shanmugam G. (2020). A Multinational, Multicentre Study on the Psychological Outcomes and Associated Physical Symptoms Amongst Healthcare Workers during COVID-19 Outbreak. Brain Behav. Immun..

[41] Hummel S., Oetjen N., Du J., Posenato E., Resende de Almeida R.M., Losada R., Ribeiro O., Frisardi V., Hopper L., Rashid A. (2021). Mental Health Among Medical Professionals During the COVID-19 Pandemic in Eight European Countries: Cross-sectional Survey Study. J. Med. Internet Res..

[42] Pašić A., Štraus S., Smajić E., Begović E., Haxhibeqiri-Karabdić I., Spasojević N. (2022). Psychosocial Influence of COVID-19 on Healthcare Workers. Med. Glas..

[43] Vagni M., Maiorano T., Giostra V., Pajardi D. (2021). Protective Factors Against Emergency Stress and Burnout in Healthcare and Emergency Workers during Second Wave of COVID-19. Soc. Sci..

[44] Satty T., Ramgopal S., Elmer J., Mosesso V.N., Martin-Gill C. (2021). EMS Responses and Non-Transports during the COVID-19 Pandemic. Am. J. Emerg. Med..

[45] Douplat M., Termoz A., Subtil F., Haesebaert J., Jacquin L., Durand G., Potinet V., Hernu R., Nohales L., Mazza S. (2022). Changes Over Time in Anxiety, Depression, and Stress Symptoms among Healthcare Workers in French Emergency Departments during the First COVID-19 Outbreak. J. Affect. Disord..

[46] Solis E.C., Van Hemert A.M., Carlier I.V.E., Wardenaar K.J., Schoevers R.A., Beekman A.T.F., Penninx B.W.J.H., Giltay E.J. (2021). The 9-Year Clinical Course of Depressive and Anxiety Disorders: New NESDA Findings. J. Affect. Disord..

[47] Lam M.K., Lam L.T., Butler-Henderson K., King J., Clark T., Slocombe P., Dimarco K., Cockshaw W. (2022). Prescribing Behavior of Antidepressants for Depressive Disorders: A Systematic Review. Front. Psychiatry.

[48] Maciaszek J., Lenart M., Misiak B., Grzebieluch J., Gawłowski P., Ciułkowicz M., Łuc D., Szcześniak D., Rymaszewska J. (2021). Unknown Enemy and Psychopathological Responses: A Cross-Sectional Nationwide Study Assessing the Knowledge about COVID-19. Front. Psychiatry.

[49] Babicki M., Szewczykowska I., Mastalerz-Migas A. (2021). The Mental Well-Being of Health Care Workers during the Peak of the COVID-19 Pandemic-A Nationwide Study in Poland. Int. J. Environ. Res. Public Health.

[50] Ministerio de Sanidad, Consumo y Bienestar Social (2019). Encuesta Nacional de Salud ENSE, España 2017. Serie Informes Monográficos #1—Salud Mental.

[51] Dantas E.S.O., Araújo Filho J.D., Silva G.W.D.S., Silveira M.Y.M., Dantas M.N.P., Meira K.C. (2021). Factors Associated with Anxiety in Multiprofessional Health Care Residents during the COVID-19 Pandemic. Rev. Bras. Enferm..

[52] Mausz J., Donnelly E.A., Moll S., Harms S., McConnell M. (2022). Mental Disorder Symptoms and the Relationship with Resilience among Paramedics in a Single Canadian Site. Int. J. Environ. Res. Public Health.

[53] Kunzler A.M., Röthke N., Günthner L., Stoffers-Winterling J., Tüscher O., Coenen M., Rehfuess E., Schwarzer G., Binder H., Schmucker C. (2021). Mental Burden and Its Risk and Protective Factors during the Early Phase of the SARS-CoV-2 Pandemic: Systematic Review and Meta-Analyses. Glob. Health.

[54] Khanal P., Devkota N., Dahal M., Paudel K., Joshi D. (2020). Mental Health Impacts among Health Workers during COVID-19 in a Low Resource Setting: A Cross-Sectional Survey from Nepal. Global Health.

[55] Bernales-Turpo D., Quispe-Velasquez R., Flores-Ticona D., Saintila J., Ruiz Mamani P.G., Huancahuire-Vega S., Morales-García M., Morales-García W.C. (2022). Burnout, Professional Self-Efficacy, and Life Satisfaction as Predictors of Job Performance in Health Care Workers: The Mediating Role of Work Engagement. J. Prim. Care Community Health.

[56] Şahin M.K., Aker S., Şahin G., Karabekiroğlu A. (2020). Prevalence of Depression, Anxiety, Distress and Insomnia and Related Factors in Healthcare Workers During COVID-19 Pandemic in Turkey. J. Community Health.

[57] Unal M., Yilmaz A., Yilmaz H., Tasdemir G.Y., Uluturk M., Kemanci A., Senol H., Altan B., Ozen M., Seyit M. (2022). The Impact of COVID-19 on Social Support Perception and Stress of Prehospital Care Providers. Australas. Emerg. Care.

[58] Aragonès E., Cura-González I.D., Hernández-Rivas L., Polentinos-Castro E., Fernández-San-Martín M.I., López-Rodríguez J.A., Molina-Aragonés J.M., Amigo F., Alayo I., Mortier P. (2022). Psychological Impact of the COVID-19 Pandemic on Primary Care Workers: A Cross-Sectional Study. Br. J. Gen. Pract..

[59] López-Atanes M., Pijoán-Zubizarreta J.I., González-Briceño J.P., Leonés-Gil E.M., Recio-Barbero M., González-Pinto A., Segarra R., Sáenz-Herrero M. (2021). Gender-Based Analysis of the Psychological Impact of the COVID-19 Pandemic on Healthcare Workers in Spain. Front. Psychiatry.

[60] Chinvararak C., Kerdcharoen N., Pruttithavorn W., Polruamngern N., Asawaroekwisoot T., Munsukpol W., Kirdchok P. (2022). Mental Health among Healthcare Workers During COVID-19 Pandemic in Thailand. PLoS ONE.

[61] Jones S., Nagel C., McSweeney J., Curran G. (2018). Prevalence and Correlates of Psychiatric Symptoms among First Responders in a Southern State. Arch. Psychiatr. Nurs..

[62] Zhu Z., Xu S., Wang H., Liu Z., Wu J., Li G., Miao J., Zhang C., Yang Y., Sun W. (2020). COVID-19 in Wuhan: Sociodemographic Characteristics and Hospital Support Measures Associated with the Immediate Psychological Impact on Healthcare Workers. EClinicalMedicine.

[63] Sobregrau P., Andreu C., Carreño M., Donaire A., Rumià J., Boget T., Bargalló N., Setoain X., Roldan P., Conde-Blanco E. (2021). Psychiatric Disorders in Patients with Resistant Temporal Lobe Epilepsy Two Years after Undergoing Elective Surgery. A Longitudinal Study. Epilepsy Behav..

[64] Zhu J., Sun L., Zhang L., Wang H., Fan A., Yang B., Li W., Xiao S. (2020). Prevalence and Influencing Factors of Anxiety and Depression Symptoms in the First-Line Medical Staff Fighting Against COVID-19 in Gansu. Front. Psychiatry.

[65] Sangrà P.S., Ribeiro T.C., Esteban-Sepúlveda S., Pagès E.G., Barbeito B.L., Llobet J.A., Moya-Prats J.L.P., Pérez L.P., Mir S.A. (2022). Mental Health Assessment of Spanish Frontline Healthcare Workers during the SARS-CoV-2 Pandemic. Med. Clin..

[66] Gil García E., Romo Avilés N., Poo Ruiz M., Meneses Falcón C., Markez Alonso I., Vega Fuente A. (2005). Gender and Psychiatric Drugs: A Qualitative Study to Find the Views of Prescription-Fillers. Aten. Primaria.

[67] Velasco Arías S. (2009). Recomendaciones para la Práctica Clínica con Enfoque de Género.

[68] Urbanos-Garrido R. (2016). Inequality in Access to Health Care Services. Policy Recommendations Aimed at Achieving Equity. Gac. Sanit..

[69] Matud Aznar M.P., Garía Pérez L., Bethencourt Pérez J.M., Rodríguez-Wangüemert C. (2017). Gender and the Use of Anxiolytic and Hypnotic Frugs in Spain. J. Fem. Gend. Women Stud..

[70] Sanford J., Agrawal A., Miotto K. (2021). Psychological Distress among Women Healthcare Workers: A Health System’s Experience Developing Emotional Support Services during the COVID-19 Pandemic. Front. Glob. Womens Health.

[71] Bae S.Y., Yoon H.J., Kim Y., Kim J. (2022). Posttraumatic Stress Disorder and Related Factors among Nurses Working during the COVID-19 Pandemic. J. Nurs. Manag..

[72] Li G., Miao J., Wang H., Xu S., Sun W., Fan Y., Zhang C., Zhu S., Zhu Z., Wang W. (2020). Psychological Impact on Women Health Workers Involved in COVID-19 Outbreak in Wuhan: A Cross-Sectional Study. J. Neurol. Neurosurg. Psychiatry.

[73] Di Tella M., Romeo A., Benfante A., Castelli L. (2020). Mental Health of Healthcare Workers during the COVID-19 Pandemic in Italy. J. Eval. Clin. Pract..

[74] Romero C.S., Delgado C., Catalá J., Ferrer C., Errando C., Iftimi A., Benito A., de Andrés J., Otero M. (2022). COVID-19 Psychological Impact in 3109 Healthcare Workers in Spain: The PSIMCOV Group. Psychol. Med..

[75] Huang Y., Zhao N. (2020). Generalized Anxiety Disorder, Depressive Symptoms and Sleep Quality during COVID-19 Outbreak in China: A Web-Based Cross-Sectional Survey. Psychiatry Res..

[76] Yildirim T.T., Atas O., Asafov A., Yildirim  K., Balibey H. (2020). Psychological Status of Healthcare Workers during the Covid-19 Pandemic. J. Coll. Physicians Surg. Pak..

[77] Lin Y.P., Chan L.Y.C., Chan E.Y. (2022). Tenacious Team, Precarious Patient: A Phenomenological Inquiry into Interprofessional Collaboration during ICU Resuscitations. J. Adv. Nurs..

[78] Solomou I., Constantinidou F. (2020). Prevalence and Predictors of Anxiety and Depression Symptoms during the COVID-19 Pandemic and Compliance with Precautionary Measures: Age and Sex Matter. Int. J. Environ. Res. Public. Health.

[79] Cai W., Lian B., Song X., Hou T., Deng G., Li H. (2020). A Cross-Sectional Study on Mental Health among Health Care Workers during the Outbreak of Corona Virus Disease 2019. Asian J. Psychiatr..

[80] Birditt K.S., Fingerman K.L., Almeida D.M. (2005). Age Differences in Exposure and Reactions to Interpersonal Tensions: A Daily Diary Study. Psychol. Aging.

[81] Lai J., Ma S., Wang Y., Cai Z., Hu J., Wei N., Wu J., Du H., Chen T., Li R. (2020). Factors Associated with Mental Health Outcomes Among Health Care Workers Exposed to Coronavirus Disease 2019. JAMA Netw. Open.

[82] Carmassi C., Foghi C., Dell’Oste V., Cordone A., Bertelloni C.A., Bui E., Dell’Osso L. (2020). PTSD Symptoms in Healthcare Workers Facing the Three Coronavirus Outbreaks: What Can We Expect after the COVID-19 Pandemic. Psychiatry Res..

[83] Cai H., Tu B., Ma J., Chen L., Fu L., Jiang Y., Zhuang Q. (2020). Psychological Impact and Coping Strategies of Frontline Medical Staff in Hunan Between January and March 2020 during the Outbreak of Coronavirus Disease 2019 (COVID-19) in Hubei, China. Med. Sci. Monit..

[84] Martínez-Caballero C.M., Cárdaba-García R.M., Varas-Manovel R., García-Sanz L.M., Martínez-Piedra J., Fernández-Carbajo J.J., Pérez-Pérez L., Madrigal-Fernández M.A., Barba-Pérez M.Á., Olea E. (2021). Analyzing the Impact of COVID-19 Trauma on Developing Post-Traumatic Stress Disorder among Emergency Medical Workers in Spain. Int. J. Environ. Res. Public Health.

[85] Amro T.M., Arcos González P., Montero Viñuales E., Castro Delgado R. (2022). Impact of COVID-19 Pandemic on Stress and Burnout Levels amongst Emergency Medical Technicians: A Cross-Sectional Study in Spain. Ann. Med..

[86] Luo M., Guo L., Yu M., Jiang W., Wang H. (2020). The Psychological and Mental Impact of Coronavirus Disease 2019 (COVID-19) on Medical Staff and General Public—A Systematic Review and Meta-Analysis. Psychiatry Res..

[87] Zwack J., Schweitzer J. (2013). If Every Fifth Physician is Affected by Burnout, What About the Other Four? Resilience Strategies of Experienced Physicians. Acad Med..

[88] Ilczak T., Rak M., Ćwiertnia M., Mikulska M., Waksmańska W., Krakowiak A., Bobiński R., Kawecki M. (2021). Predictors of Stress among Emergency Medical Personnel During the COVID-19 Pandemic. Int. J. Occup. Med. Environ. Health.

[89] Karasu F., Öztürk Çopur E., Ayar D. (2022). The Impact of COVID-19 on Healthcare Workers’ Anxiety Levels. Z. Gesundh Wiss.

[90] Usul E., Şan I., Bekgöz B. (2020). The Effect of the COVID-19 Pandemic on the Anxiety Level of Emergency Medical Eervices Professionals. Psychiatr. Danub..

[91] Skoda E.M., Teufel T., Stang A., Jöckel K.H., Junne F., Weismüller B., Hetkamp M., Musche V., Kohler H., Dörrie N. (2020). Psychological Burden of Healthcare Professionals in Germany during the Acute Phase of the COVID-19 Pandemic: Differences and Similarities in the International Context. J. Public Health.

[92] Andrew E., Nehme Z., Stephenson M., Walker T., Smith K. (2021). The Impact of the COVID-19 Pandemic on Demand for Emergency Ambulances in Victoria, Australia. Prehosp. Emerg. Care..

[93] Wojtysiak K., Zielińska-Więczkowska H. (2022). Work in Stressful Conditions in Medical Emergency System during the COVID-19 Pandemic. Med. Pr..

[94] Li T.M., Pien L.C., Kao C.C., Kubo T., Cheng W.J. (2022). Effects of Work Conditions and Organizational Strategies on Nurses’ Mental Health during the COVID-19 Pandemic. J. Nurs. Manag..

[95] Blanchard J., Li Y., Bentley S.K., Lall M.D., Messman A.M., Liu Y.T., Diercks D.B., Merritt-Recchia R., Sorge R., Warchol J.M. (2022). The Perceived Work Environment and Well-being: A Survey of Emergency Health Care Workers during the COVID-19 Pandemic. Acad. Emerg. Med..

[96] Malinowska-Lipień I., Wadas T., Gabryś T., Kózka M., Gniadek A., Brzostek T., Squires A. (2022). Evaluating Polish Nurses’ Working Conditions and Patient Safety during the COVID-19 Pandemic. Int. Nurs. Rev..

[97] Salari N., Khazaie H., Hosseinian-Far A., Khaledi-Paveh B., Kazeminia M., Mohammadi M., Shohaimi S., Daneshkhah A., Eskandari S. (2020). The Prevalence of Stress, Anxiety and Depression within Front-Line Healthcare Workers Caring for COVID-19 Patients: A Systematic Review and Meta-Regression. Hum. Resour. Health.

[98] Walton M., Murray E., Christian M.D. (2020). Mental Health Care for Medical Staff and Affiliated Healthcare Workers during the COVID-19 Pandemic. Eur. Heart J. Acute Cardiovasc. Care.

[99] David E., DePierro J.M., Marin D.B., Sharma V., Charney D.S., Katz C.L. (2022). COVID-19 Pandemic Support Programs for Healthcare Workers and Implications for Occupational Mental Health: A Narrative Review. Psychiatr Q..

[100] Buselli R., Corsi M., Veltri A., Baldanzi S., Chiumiento M., Lupo E.D., Marino R., Necciari G., Caldi F., Foddis R. (2021). Mental Health of Health Care Workers (HCWs): A Review of Organizational Interventions Put in Place by Local Institutions to Cope with New Psychosocial Challenges Resulting from COVID-19. Psychiatry Res..

